# Utility of power Doppler ultrasonography for detecting forefoot bursae in early rheumatoid arthritis: A case report

**DOI:** 10.1097/MD.0000000000013295

**Published:** 2018-12-21

**Authors:** Yushiro Endo, Tomohiro Koga, Mizuna Eguchi, Momoko Okamoto, Sosuke Tsuji, Ayuko Takatani, Toshimasa Shimizu, Remi Sumiyoshi, Takashi Igawa, Shin-ya Kawashiri, Naoki Iwamoto, Kunihiro Ichinose, Mami Tamai, Hideki Nakamura, Tomoki Origuchi, Atsushi Kawakami

**Affiliations:** Department of Immunology and Rheumatology, Unit of Advanced Preventive Medical Sciences, Nagasaki University Graduate School of Biomedical Sciences, Nagasaki, Japan.

**Keywords:** forefoot bursitis, intermetatarsal bursitis, power Doppler ultrasonography, rheumatoid arthritis

## Abstract

**Rationale:**

Power Doppler ultrasonography (PDUS) in musculoskeletal ultrasound (MSUS) is a sensitive and reliable method for the assessment of rheumatoid arthritis (RA). The association between ultrasound-detectable forefoot bursae and the development of RA has gained attention. However, a few studies have evaluated the utility of PDUS for examining forefoot bursae in early RA. We report the case of an RA patient who developed reduced foot mobility and had detectable intermetatarsal bursitis with remarkable power Doppler (PD) signals in MSUS at the onset of RA.

**Patient concerns:**

A 40-year-old Japanese woman diagnosed with palindromic rheumatism visited our department due to sustained forefoot pain and difficulty walking. The levels of both rheumatoid factor (RF) and anticitrullinated protein antibody (ACPA) were high. She had opening toes with swelling in metatarsophalangeal (MTP) joints. PDUS showed intermetatarsal bursitis with mild MTP synovitis.

**Diagnoses:**

We diagnosed RA by comprehensive judgment based on the 2010 American College of Rheumatology and European League Against Rheumatism classification criteria for RA.

**Interventions:**

We administered 6.0 mg/wk of methotrexate (MTX) and 2.0 mg/d of prednisolone (PSL) followed by an increase of MTX to 10 mg/wk.

**Outcomes:**

After those treatments, the patient's symptoms showed improvement. As of this writing, the patient's remission has been maintained for >2 months.

**Lessons:**

Her case suggests that PDUS is useful for the detection of forefoot bursitis, and the detection of forefoot bursitis by PDUS may provide the opportunity to make an early diagnosis of RA.

## 1 Introduction

The metatarsophalangeal (MTP) joints frequently exhibit synovitis in early rheumatoid arthritis (RA) patients.^[1]^ Although RA patients sometimes suffer from reduced mobility due to foot pain and dysfunction, clinical examinations of feet may not be performed routinely because the tools that are commonly used to measure disease activity omit the feet and ankle joints.^[2]^ The symptoms of foot pain and dysfunction were previously thought to be caused by synovitis and damage in the MTP joints,^[1,3]^ but as detailed observations with improved imaging methods became possible, these symptoms have been recognized to be caused by flexor tenosynovitis and bursae within the forefoot in addition to synovitis and damage in the MTP joints.^[4–7]^

Power Doppler ultrasonography (PDUS) in musculoskeletal ultrasound (MSUS) is a sensitive and reliable method for the assessment of RA.^[8]^ The association between ultrasound-detectable forefoot bursae and the development of RA has gained attention.^[2,4–6]^ However, few studies have evaluated the utility of PDUS for examining forefoot bursae in early RA. We herein report the case of an RA patient who developed reduced foot mobility and had detectable intermetatarsal bursitis with remarkable power Doppler (PD) signals in MSUS at the onset of RA.

## 2 Case report

In May 2017, a 40-year-old Japanese woman presented arthralgia at the left wrist and visited a local orthopedic department. Laboratory investigations showed that her levels of C-reactive protein (CRP) and rheumatoid factor (RF) were within the normal ranges at 0.14 mg/dL and 9.0 IU/mL, respectively, but her level of anticitrullinated protein antibody (ACPA) was high at 101 U/mL. She was then referred to our Immunology and Rheumatology Department for the evaluation of RA.

At the patient's first visit to our department, her arthralgia had already disappeared and she had no clinical symptoms. Imaging findings such as X-ray at both hands and feet, the MSUS assessment of peripheral upper limbs, and magnetic resonance imaging (MRI) at both hands showed no findings of synovitis, tenosynovitis, or damage of the joints. After that visit, the patient suffered from recurrent arthralgia lasting for 1 to 2 weeks at the joints of both wrists, elbows, or hips, and the level of RF became positive. However, she showed no findings of sustained arthritis. We thus finally diagnosed palindromic rheumatism.

**Figure 1:**
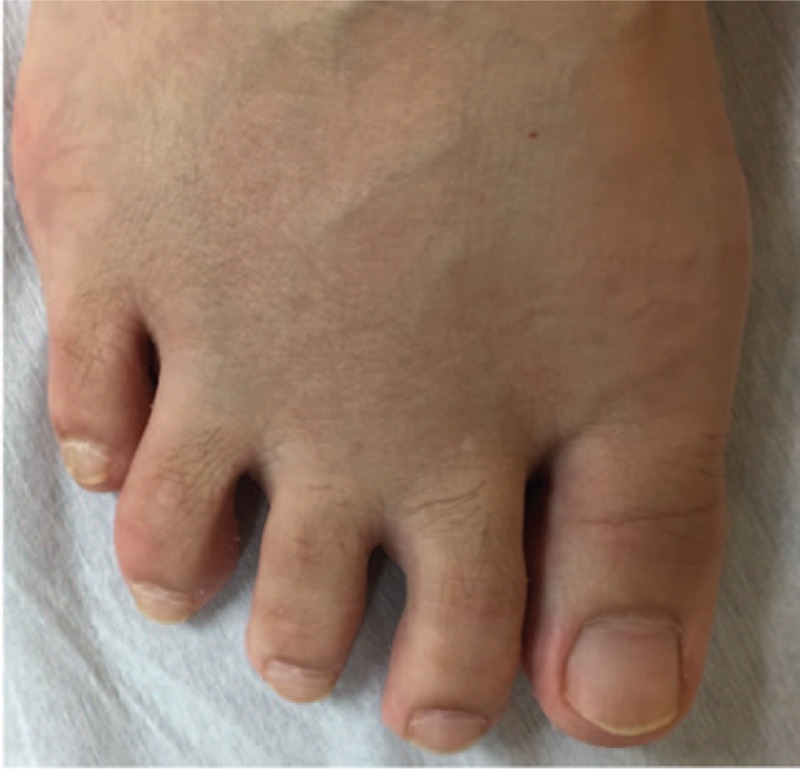
Opening toes in the 2nd to 3rd and 3rd to 4th toes of the patient's right foot with swelling in the 2nd to 4th MTP joints. MTP = metatarsophalangeal.

In January 2018, the patient presented sustained arthralgia at toes of both feet and visited our department again. On physical examination, swelling and tenderness in the 2nd to 4th MTP joints of both feet were observed, but MSUS of both feet showed no findings of synovitis or tenosynovitis. However, during the follow-up, the patient had difficulty walking due to the forefoot pain, and she thus came to our department again in March 2018. On physical examination, opening at both the 2nd to 3rd and 3rdto 4th toes with swelling and tenderness in the 2nd to 4th MTP joints of both feet were newly observed (Fig. 1). Laboratory investigations showed the following results: white blood cell count 8300/μL (neutrophils 69.2%), hemoglobin 12.4 g/dL, platelet 28.9 × 10^4^/μL, CRP 0.18 mg/dL. antinuclear antibody 80 times (homogenous: 80 times, speckled: 80 times), erythrocyte sedimentation rate (ESR) 12 mm/h, RF 123.5 IU/mL, ACPA 461.3 U/mL. The serum complement level was normal. X-ray examinations of both the hands and feet showed no findings of bone erosion or joint space narrowing.

**Figure 2:**
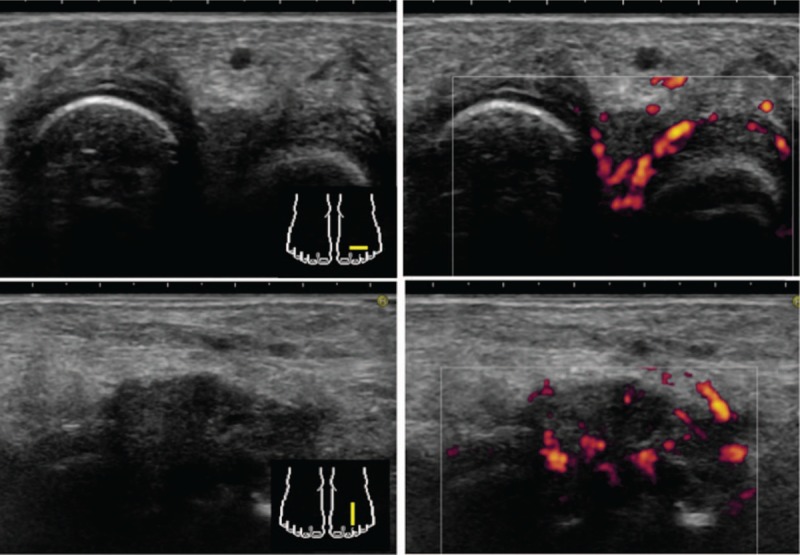
Cross and longitudinal section of the MSUS showed intermetatarsal bursitis with remarkable PD signals in the 2rd to 3th toes of left feet with mild synovitis in the left 3th MTP joint. MSUS = musculoskeletal ultrasound, MTP = metatarsophalangeal, PD = power Doppler.

We suspected the presence of intermetatarsal bursitis based on the findings of opening between toes. Accordingly, we performed another MSUS assessment, which revealed showing intermetatarsal bursitis with remarkable PD signals in both the 2nd to 3rd and 3rd to 4th toes with mild synovitis in the right 1st and left 3th MTP joints (Fig. 2). The patient did not have a history of mechanical stress to her forefoot that would trigger intermetatarsal bursitis. The patient's findings fulfilled the 2010 American College of Rheumatology and European League Against Rheumatism classification criteria for RA^[9]^ due to one small joint involvement, high-positive RF and ACPA, and duration of her symptoms for more than 6 weeks. Although the findings and numbers of her synovitis were mild and few respectively, we diagnosed RA by comprehensive judgment considering intermetatarsal bursitis with remarkable PD signals and administered 6.0 mg/wk of methotrexate (MTX). Immediately after the initiation of the treatment, the patient presented swelling and tenderness in the 2nd MCP joint. We added on 2.0 mg/d of prednisolone (PSL) and increased MTX to 10 mg/wk. After those treatments, the patient's symptoms showed improvement. As of this writing, the patient's remission has been maintained for >2 months.

## 3 Discussion

Although a clinical examination is the most basic and important method for the diagnosis and monitoring of RA, a clinical examination alone is not enough itself because of its low accuracy and reproducibility.^[10]^ The detection of cartilage loss and bone erosion by radiography is also a traditional and essential imaging method for the diagnosis and monitoring of RA,^[11]^ but it is not sufficiently sensitive for the diagnosis of RA, especially at the early stage.^[12,13]^ There is increasing evidence that MRI has a high diagnostic value for RA and can accurately detect inflammatory and destructive joint changes in RA patients.^[14–18]^

MSUS has been drawing attention as a new imaging method for the evaluation of joints in patients with rheumatic disease.^[19]^ MSUS is a valuable clinical tool that is comparable to and more accessible than MRI in the assessment of soft tissues in RA patients.^[5,11]^ MSUS is also useful for the detection of not only articular synovitis but also tenosynovitis and bursitis in RA patients.^[20,21]^

Forefoot bursae is one of the causes of metatarsalgia.^[22]^ Forefoot bursae with enlargement and inflammation can cause clinical symptoms.^[23,24]^ Depending on their location, synovial bursae within the forefoot are of 2 types: submetatarsal and intermetatarsal bursae.^[5,25]^ Both bursae can be evaluated by MSUS.^[4,6]^ Submetatarsal bursae are adventitial bursae defined as fluid-filled spaces without a synovial lining.^[26]^ They are located in the subcutaneous tissues at the level of the metatarsal heads, and they are considered mechanically derived due to chronic local overload.^[23,25]^ Conversely, the intermetatarsal bursae are anatomic bursae that have a synovial lining,^[27,28]^ and these bursae are clinically significant due to their close cohesion with the intermetatarsal neurovascular bundle.^[22,29]^ Intermetatarsal bursae with hypertrophy can appear on US as a well-defined fluid collection with hypoechoic or anechoic zones usually bulging >1 mm under the level of the metatarsal heads.^[6,30]^

Compared with a clinical examination, MSUS has more accurately detected forefoot bursae. A 2010 study showed that although no forefoot bursae were detected clinically in control subjects, 38% of the subjects had one or more bursae detectable by MSUS.^[5]^ In the same study, approx. 90% of the RA patients had one or more bursae detectable by MSUS, and the RA patients had a significantly higher prevalence of detectable bursae and a significantly larger mean number of detectable bursae per individual compared with the control subjects.^[5]^ In another study, the number of detectable forefoot bursae in control subjects was smaller than that in RA patients.^[6]^

In RA patients, forefoot bursae regress or undergo hypertrophy over time, and these changes are associated with reduced foot mobility due to foot pain and dysfunction independent of changes in overall disease activity (such as the levels of CRP and ESR, and VAS and DAS28 scores).^[4]^ An investigation of early RA patients whose mean disease duration was 1.1 years showed a significantly higher prevalence of intermetatarsal bursae detectable by MSUS compared with healthy subjects, and 24% (6/25) of the early RA patients with clinical symptoms in the forefoot showed no findings in the forefoot by MSUS except for detectable intermetatarsal bursae.^[6]^ Because our patient showed not only mild MTP synovitis but also remarkable intermetatarsal bursitis at the onset of disease, these findings as well as her serological results contributed to a definite diagnosis of RA at the early stage. It is thus important to evaluate not only synovitis and damage in MTP joints and flexor tenosynovitis but also forefoot bursitis in patients with RA or suspected RA, by imaging methods such as MSUS.

To the best of our knowledge, there have been no investigations evaluating the inflammation of forefoot bursae in RA patients using PD signaling on MSUS or reports that track such inflammation with PD signals after the initiation of treatment. As mentioned above, forefoot bursae detectable by MSUS—with or without PD signals—are also not rare in healthy individuals. Although it is not yet established how the presence of a PD signal or its intensity in forefoot bursae can contribute to the diagnosis of RA, it might be informative to use PDUS to evaluate the inflammation of detectable forefoot bursae to determine whether or not a patient has inflammatory disease. Although we suspect that detectable intermetatarsal bursitis with a PD signal on MSUS are not rare at the onset of RA, our speculation remains to be tested. Further investigation is needed to clarify the association between detectable intermetatarsal bursitis with PD signals in MSUS and the development of RA at the early stage.

In conclusion, we successfully treated a patient with RA who developed reduced foot mobility and had detectable intermetatarsal bursitis with remarkable PD signals in MSUS at the onset of RA. RA patients have a higher prevalence of forefoot bursae at the early stage. We emphasize the necessity of evaluating not only synovitis and damage in MTP joints and flexor tenosynovitis but also forefoot bursae in individuals with RA or suspected RA who have forefoot symptoms. The present case suggests that MSUS is useful for the detection of forefoot bursitis, and the detection of forefoot bursitis with a PD signal by MSUS may help make an early diagnosis of RA.

## Author contributions

**Supervision:** Tomohiro Koga, Mizuna Eguchi, Momoko Okamoto, Sosuke Tsuji, Ayuko Takatani, Toshimasa Shimizu, Remi Sumiyoshi, Takashi Igawa, Shin-ya Kawashiri, Naoki Iwamoto, Kunihiro Ichinose, Mami Tamai, Hideki Nakamura, Tomoki Origuchi, Atsushi Kawakami.

**Writing – original draft:** Yushiro Endo.

**Writing – review & editing:** Tomohiro Koga.
